# Dislocation rates of postoperative airway exchange catheters - a prospective case series of 200 patients

**DOI:** 10.1186/s12871-019-0723-9

**Published:** 2019-04-11

**Authors:** Fredy-Michel Roten, Richard Steffen, Maren Kleine-Brueggeney, Robert Greif, Marius Wipfli, Andreas Arnold, Henrik Fischer, Lorenz Theiler

**Affiliations:** 1 Department of Anesthesiology and Pain Therapy, Bern University Hospital and University of Bern, CH-3010 Bern, Switzerland; 2 Department of Anaesthesia, Evelina London Children’s Hospital, Guys and St. Thomas’ NHS Foundation Trust, London, SE1 7EH UK; 3 Department of Anaesthesiology and Pain Therapy, Lindenhofspital, CH-3011 Bern, Switzerland; 4 Department of Otorhinolaryngology, Head and Neck Surgery, Bern University Hospital and University of Bern, CH-3010 Bern, Switzerland; 5 Medical School, Sigmund Freud University, Kelsenstraße 2, A -1030 Vienna, Austria

**Keywords:** Airway, Extubation, Intubation, Airway exchange catheter, Oral, Nasal, Postoperative, Dislocation

## Abstract

**Background:**

The dislocation rate of oral versus nasal airway exchange catheters (AEC) in the postoperative care unit (PACU) are unknown. Our aim was to establish dislocation rates and to assess the usefulness of waveform capnography to detect dislocation.

**Methods:**

In this non-randomized, prospective observational trial at the University Hospital Bern, Switzerland, we included 200 patients admitted to PACU after extubation via AEC, having provided written informed consent. The study was approved by the local ethical committee. AEC position was assessed by nasal fiberoptic endoscopy at beginning of PACU stay and before removal of the AEC. Capnography was continuously recorded via the AEC. Additional measurements included retching and coughing of the patient, and re-intubation, if necessary.

**Results:**

Data from 182 patients could be evaluated regarding dislocation. Overall dislocation rate was not different between oral and nasal catheters (7.2% vs. 2.7%, *p* = 0.16). Retching was more often noted in oral catheters (26% vs. 8%, *p* < 0.01). Waveform capnography was unreliable in predicting dislocation (negative predictive value 17%). Re-intubation was successful in all five of the nine re-intubations where an AEC was still in situ. In four patients, the AEC was already removed when re-intubation became necessary, and re-intubation failed once, with a front of neck access as a rescue maneuver.

**Conclusions:**

We found no difference in dislocation rate between nasal and oral position of an airway exchange catheter. However, nasal catheters seemed to be tolerated better. In the future, catheters like the staged extubation catheter may further increase tolerance.

**Trial registration:**

The study was registered in a clinical study registry (ISRCTN 96726807) on 10/06/2010.

## Background

Tracheal extubation requires as much dedication and attention as tracheal intubation, but this is often neglected, and thus adverse events during extubation are frequent. The British National Audit Project 4 showed that one sixth of all reported cases of serious adverse events occurred upon emergence or during recovery from anesthesia [1]. Likewise, complications during extubation are potentially harmful, with a reported mortality rate of 5% and a 13% rate of severe adverse outcomes with extubation failure related to general anesthesia [1]. Hence, the use of a staged extubation plan is recommended, which may include an airway exchange catheter (AEC) [2, 3]. The AEC was initially designed as an airway exchange catheter, not as an extubation catheter, hence its name. The AEC is a device, designed to maintain access to the airway after extubation to facilitate reintubation. As such, the rate of reported complications during exchange of a tracheal tube is quite high. A retrospective report in 2013 reported a failure rate of 9.3% (39.3% when exchanging to a double lumen tube) and the airway injury rate was 7.8% with a 1.5% rate of pneumothorax [4]. In that study, difficult tube exchange was encountered in 6 of 8 patients with pneumothorax.

When used as a back-up device for extubation, the AEC were successful in 7 of the 9 cases (78%) when patient had to be re-intubated postoperatively [4]. In a study in ICU, the AEC showed an overall success rate of 92% (47 of 51) and a first-pass success rate of 87% [5]. Three out of 51 (6%) patients could not be intubated even after multiple attempts, and dislocation of the AEC may have been a reason for this. The report does not indicate whether these patients had oral or nasal AECs, which might have made a difference.

Based on these studies, it is unclear whether a nasally or orally placed AEC would show a lower dislocation rate and which position would be better tolerated by the patient.

We therefore prospectively evaluated the position of the AEC in patients admitted to the PACU who were extubated via an AEC. We expected the dislocation rate of the AEC to be different, depending on nasal versus oral position.

## Methods

The local ethics committee approved this prospective observational study (Kantonale Ethikkommission KEK, Bern, Switzerland, reference number 060/10) and the study was registered in a clinical study registry (ISRCTN 96726807). For this observational, nonrandomized quality control study, we prospectively included two hundred adult patients (> 18 years old) admitted to the PACU who were extubated via an AEC (Cook Airway Exchange Catheter, Cook Medical Inc., Bloomington, IN, USA) and who gave written informed consent to use their data obtained during the PACU stay. There were no other exclusion criteria defined. As the decision for an AEC was made by the attending anesthesia team in the OR, obtaining informed consent while the patient was still awake prior to surgery was not possible. Therefore, informed consent was obtained at the time of discharge from the PACU when the patient was awake, fully oriented, free of pain and with stable vital signs. If this was not the case, the patients were consented on the day after surgery on the ward or, if the patients had already left the hospital, by telephone and postal letter. The decision to place an AEC was based on clinical judgment of the attending anesthesiologist and the surgeon in the operating room who were not part of the study group and not involved in PACU care, as was the choice of nasal vs. oral placement and the size of the AEC.

At admission to the PACU, the following AEC parameters were obtained: location, size, depth from either the corner of the mouth or the nares, and indication of the AEC. Coughing and retching as part of the patient’s tolerance of the AEC were also noted throughout the PACU stay. Other recorded parameters included demographic parameters, the real and the planned period the AEC remained in situ, as well as side effects.

End-tidal carbon dioxide was measured and recorded continuously as waveform capnography until removal of the AEC using a Philips Sidestream™ system (Philips Medizin Systeme, Böblingen GmbH, Germany) by connecting the CO_2_ sample line (Straight Sample Line H, Philips) to the adapter on the AEC. We assumed that the ability to measure CO_2_ would reflect correct position.

The position of the AEC was verified by the attending anesthesiologist of the PACU with a flexible 2.8 mm nasopharyngoscope (Karl Storz GmbH & Co. KG, Tuttlingen, Germany) on arrival to the PACU and before removal of the AEC. Finally, the incidence of re-intubation was recorded along with the success of re-intubation.

### Statistical analysis

Our primary outcome parameter was overall dislocation rate. Secondary outcomes were patients’ characteristics, size of AEC, depth of AEC and position on arrival at PACU, length of stay of AEC and side effects (coughing and retching) and reintubation rate.

We hypothesized that nasally placed AECs remain significantly more often in the correct position in the trachea up until the time of removal, and that the difference of correct position compared to oral AECs would be at least 10%. H_0_ = Dislocation rate oral – Dislocation rate nasal < 10%.

The sample size was based on a pilot observation which showed 3 of 30 (10%) dislocated AEC in the oral group vs. 0 of 30 (0%) dislocated AEC in the nasal group. To reach a power of 80% with a one-sided alpha of *p* = 0.05, a total of 158 patients are necessary. To compensate for missing data and drop-outs, we decided to include 200 patients.

Binary data were analyzed by Chi square test, or by Fisher’s exact test if more than 20% of expected values were below 5. Ordinal data were evaluated using Kruskal-Wallis test. Continuous data were checked for normality by Q-Q plots and Shapiro-Wilk test. Normally distributed data were analyzed by Student’s t-test, otherwise Mann-Whitney u-test was used.

Binary data are presented as numbers (%), continuous data as mean ± standard deviation (SD) if normally distributed, and otherwise as median with interquartile range (25th to 75th percentile). A probability of *p* < 0.05 was considered statistically significant. Data were analyzed using stata V.15.1 (StataCorp™, College Station, TX, USA).

## Results

We prospectively included 200 patients who were admitted to the PACU after tracheal extubation via an oral or nasal AEC between December 2009 and May 2011. Two datasheets had to be dismissed because of an excess of missing data, leaving 198 patients for analysis. All patients provided written informed consent to use their data.

Seventy-four patients presented with an oral AEC in place after extubation, and 124 patients had a nasal AEC. Patients were treated predominantly in ENT, followed by orthopedics (Table 1). There was no difference in demographics between the two groups regarding sex, American Society of Anesthesiologists (ASA) class, weight, or body mass index. Patients with an oral catheter were slightly older. Most often, an 11 French catheter was used (71%, Table 2).

**Table 1:** Demographics

*n* = 198	Oral AEC *n* = 74	Nasal AEC *n* = 124	*p*-value
Female; n (%)	25 (34)	34 (27)	0.34
Surgical intervention ENT/Orthopedics/Maxillofacial/General/missing data n (%)	46/16/1/8/3 (62/22/1/11/4)	65/26/14/1/18 (52/21/11/1/15)	0.001
Age in years (mean ± SD)	63.2 ± 14.2	58.1 ± 16.1	0.03
Weight in kg (mean ± SD)	77.1 ± 17.5	74.8 ± 17.0	0.37
Height in cm (mean ± SD)	170.3 ± 8.2	172.4 ± 10.3	0.15
BMI kg m^− 2^ (mean ± SD)	26.6 ± 5.8	25.1 ± 4.8	0.05
ASA class I/ II/ III/ IV/ missing data; n (%)	5/31/33/2/3 (7/42/45/3/4)	10/41/64/4/5 (8/33/52/3/4)	0.81

Data are mean and standard deviation (SD), or numbers and percent

*AEC* Airway Exchange Catheter, *ENT* ears, nose, and throat

**Table 2:** Main Outcome Parameters

*n* = 198	Oral AEC *n* = 74	Nasal AEC *n* = 124	*p*-value
Size of AEC in French 8/ 11/ 14/ 19, n (%) *missing: 3 nasal*	0/48/22/4 (0/65/30/5)	1/71/47/2 (1/59/39/2)	0.23
Depth of AEC in cm (mean ± SD)	26.2 ± 3.3	29.3 ± 2.5	<0.001
AEC was correctly positioned on arrival PACU, yes (%)	68 (96%)	118 (98%)	0.67
95% confidence interval of correct position *missing: 3 in each group*	88.1 – 99.1%	92.9 – 99.5%	
AEC was correctly positioned until removal yes/no, n (%)	64/ 5 (93/ 7)	110/ 3 (97/ 3)	0.16
95% confidence interval of correct position *missing: 5 oral, 11 nasal*	83.9 – 97.6%	92.4 – 99.4%	
Length of stay of AEC in hours, median (IQR) min. – max.	2.5 (1.25, 4.5) 0 – 11	4 (3, 6) 0 – 19	<0.001
Patients coughing in PACU, yes n (%) *missing: 6 oral, 15 nasal*	28 (41%)	30 (28%)	0.06
Patients retching in PACU, yes n (%) *missing: 6 oral, 9 nasal*	18 (26%)	8 (8%)	0.001
Re-intubation necessary	1 (via AEC)	4 (via AEC), 4 (AEC already removed)	0.56

Data are numbers and percent, mean and standard deviation (SD) or median and interquartile range (IQR)

*AEC* Airway Exchange Catheter

### Dislocation rate and side effects

At the time of entering PACU, 4% of oral catheters and 2% of nasal catheters were already displaced as determined by nasal endoscopy. When analyzing dislocation rate, 16 datasets (5 oral AEC, 11 nasal AEC) had to be excluded because of insufficient data regarding AEC position at the time of removal. These 8% missing data represented catheters being removed without prior checking by the attending anesthesiologist (Table 2). Regarding the primary outcome parameter, overall dislocation rate, there was no significant difference between the oral and the nasal position of the catheter and thus, the null hypothesis could not be rejected (7.2% vs. 2.7%, *p* = 0.16). The odds ratio of dislocation of oral AECs vs. nasal AEC was 2.86 (95% CI: 0.66–12.39).

Interestingly, significantly more patients were retching when an oral catheter was in place compared with a nasal catheter (26% vs. 8%, *p* < 0.01). In the group with oral catheters, 41% were coughing, compared to 28% in the nasal group (*p* = 0.06). The size of the AEC did not influence retching (*p* = 0.53). Following anatomical differences between the nasal and the oral position, nasal catheters were introduced deeper compared to oral catheters (26 cm vs. 29 cm, Table 1). No serious side effects such as pneumothorax were encountered.

### Capnography as indicator of correct position

CO_2_ data from 20 catheters were incomplete and had to be excluded. In both groups, oral and nasal, there was one catheter dislodged as verified by nasal endoscopy even though CO_2_ could always be measured (oral AECs: 1 of 59 vs. nasal AECs 1 of 96). Six of 8 oral AEC and 13 of 15 nasal AEC did not show CO_2_, even though the intratracheal position was confirmed by nasal endoscopy. As a test of correct tracheal position, the presence of CO_2_ showed an overall sensitivity of 89% and a specificity of 67%. The overall positive predictive value (PPV) was 98.7% (indicating that a correct tracheal position was likely if CO_2_ present), the negative predictive value (NPV) was only 17% (waveform capnography often did not show CO_2_ even in correctly positioned catheters).

### Removal of AEC and re-intubation

**Fig. 1:**
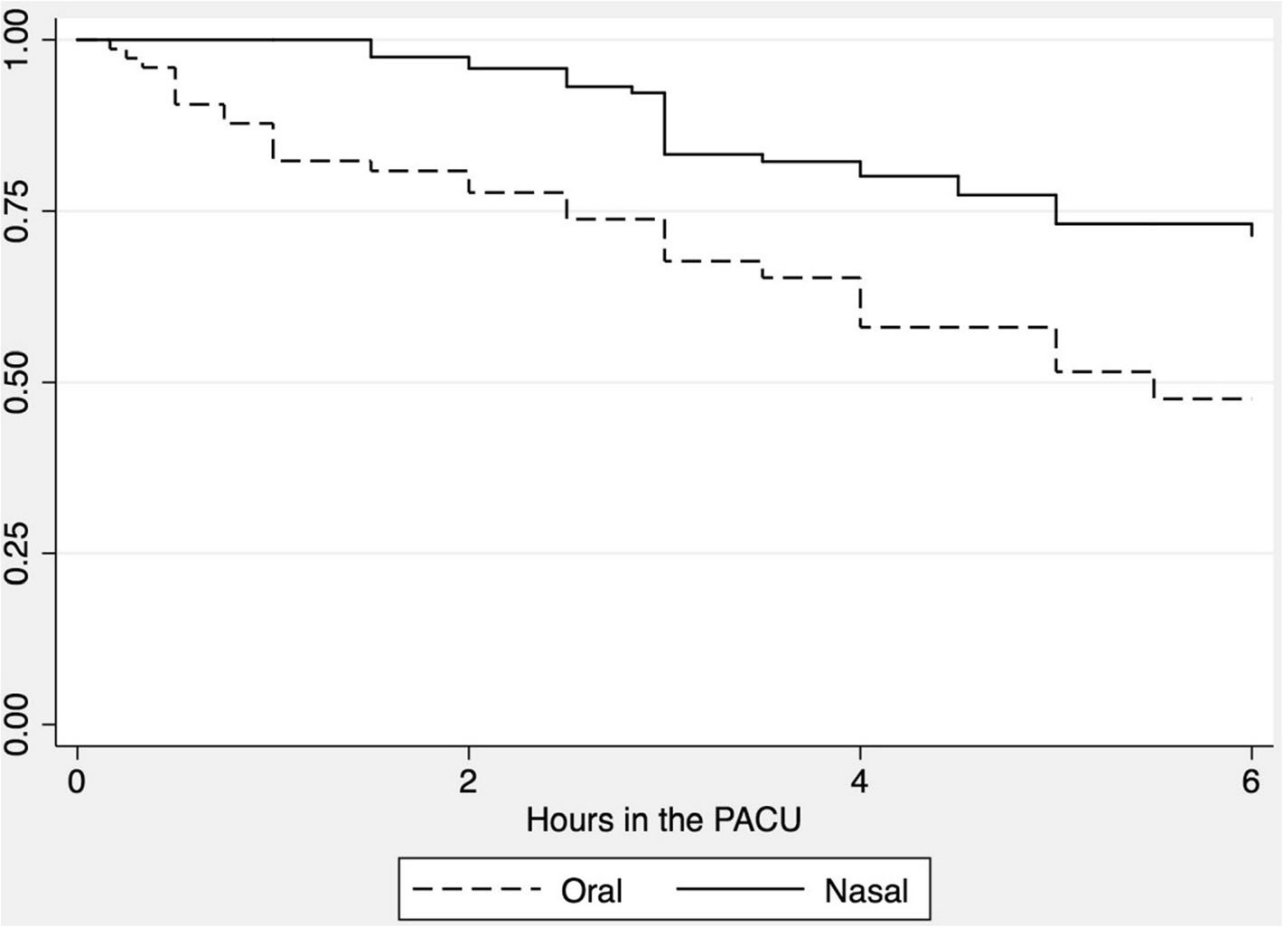
Kaplan-Meyer curve showing the ratio of catheters remaining in situ. This figure shows a Kaplan-Meyer curve of the first 6 h in the post-anesthesia care unit (PACU) showing the ratio of catheters (oral and nasal) remaining in situ

Oral catheters were removed earlier compared to nasal catheters, reflecting the plan for earlier removal of these catheters. Nevertheless, a marked drop in numbers of oral AECs in the first hour after PACU admission was noted (Fig. 1). Additionally, 3 oral catheters remained in place longer than 6 h (maximum of 11), whereas 16 nasal catheters remained in place longer than 6 h, 6 of them longer than 12 h (maximum of 19).

Of the 198 studied patients, re-intubation due to respiratory insufficiency was necessary in 9 patients (Table 2). In only 5 of these patients the AEC was still in situ: one patient in the oral group and four patients in the nasal group. All re-intubations were successful via the AEC. In four patients, re-intubation became necessary after the AEC was removed (1 to over 12 h after removal). This was successful in three patients. In one patient a surgical airway was necessary, with good outcome.

## Discussion

This prospective observational study showed that orally placed AEC tended to have a higher dislocation rate compared to nasally placed AEC (odds ratio 2.86). However, and contrary to our expectations, this finding was not statistically significant (95% confidence interval of the odds ratio was 0.66–12.39).

The non-invasive capnography proofed to be a double-edged sword and not highly reliable to verify the position of the AEC. On the one hand, presence of CO_2_ was highly suggestive of correct intratracheal position, as was reflected by the high positive predictive value of 99%. On the other hand, obstruction of the AEC lumen was frequent, which led to loss of CO_2_ reading and required additional care. Apparently, the only reliable option is to check AEC position via (nasal) flexible scope.

Based on our results of a relatively high overall dislocation rate of 4.4%, and our findings that dislocation cannot be ruled out by non-invasive means, we argue that the insertion of an AEC is not a reliable back-up for re-intubation in case of a known difficult airway. Furthermore, the chance of an esophageal dislocation should be a strong argument against the application of oxygen via the AEC, as has been pointed out by others [6–8]. Our dislocation rate of 4.4% was even smaller than reported before: A small audit in 18 patients revealed a dislocation rate of 11%, a further 11% did not tolerate the AEC [9].

Of note, the catheters were often inserted too deeply, compared to current guidelines: the mean insertion depth of oral catheters was 26.6 cm compared to a recommended maximum of 25 cm [10, 11]. Inserting AEC beyond recommended limits may lead to airway trauma and potentially death because of a (tension) pneumothorax, especially when additional oxygen is applied via the AEC [12]. Fortunately, we never encountered this complication in our study, but we also did not apply additional oxygen via the AEC. Catheters that feature a soft tip may have the potential to reduce the incidence of barotrauma and airway injury, but this has not been studied so far [13]. Either way, it is crucial to carefully avoid deep insertion of the AEC, and given the fact that others have reported complications from too deeply inserted AECs one must assume that this remains one of the main complications of AECs. A safer way may be to provide nasal oxygen during the re-intubation attempt, either low-flow [14] or high-flow nasal oxygen [15].

Oral catheters were removed earlier compared to nasal catheters, although this was frequently due to planned removal. However, the drop in AEC numbers over the first hour was more dominant in the oral group, perhaps reflecting frequent patient discomfort caused by coughing and retching with an oral AEC in place. In fact, the only statistically significant difference we could find was a higher incidence of postoperative retching in the oral group compared to nasally placed catheters (*p* < 0.01).

When looking at the re-intubation rate, a surprisingly high number of patients was re-intubated after removal of the AEC (4 out of 9). The fact that only 5 of 198 AECs were used for re-intubation also means that the use of 97.4% of the AECs (193 of 198) was unnecessary, which led to unnecessary patient discomfort, costs and potential adverse events. However, it is extremely difficult to predict which patients will require re-intubation, and for those patients who do require re-intubation a (correctly positioned) AEC can potentially be life-saving or at least avoid an emergency front of neck access. The overall re-intubation rate (9 of 198, 5%) was lower than the 8% reported earlier [16], although our data regarding re-intubation comprise only patients who were re-intubated in the PACU, not patients who were re-intubated in the operating room or patients who did not receive an AEC at all.

The use of an AEC for re-intubation in expected difficult extubation is recommended by many experts and guidelines [2, 6]. In our study, the success rate of re-intubation via AEC was 100% (5 out of 5), similar to the overall success rate of 92% (47 of 51) reported by Mort [5]. In that study, in addition to the benefit of high re-intubation success rates, the use of an AEC was associated with fewer episodes of severe hypoxemia (6% vs. 19%), of multiple intubation attempts (10% vs. 77%) and of esophageal intubation (0% vs. 18%), as pointed out in the accompanying editorial by Biro and Priebe [17]. To further increase the re-intubation success rate there is also the possibility to use an Aintree Intubation Catheter (Cook Medical Inc., Bloomington, IN, USA) in order to reduce the gap between a small AEC and the tracheal tube [18].

Our study also confirms the necessity of the presence of an adequate anesthesia service for high risk patients, even many hours postoperatively. As was described before, re-intubation can become necessary many hours postoperatively [5, 16]. Almost half of all re-intubations in our study occurred after removal of the AEC, between 1 and over 12 h after removal. To further improve the tolerance to the AEC, a wire-based AEC is available (Cook Staged Extubation Catheter™, Cook Medical Inc., Bloomington, IN, USA). A small preliminary study suggested high tolerance, as 17 of 23 patients (73%) tolerated the wire for 4 h, although “tolerated” was not further quantified [19]. Success rate and dislocation rate have not been proven to be different from the conventional AEC: Nasal endoscopy was performed in 11 of these patients and revealed one wire dislocated to the esophagus, which would correspond to a dislocation rate of 9%. In another recent small study, Furyk et al. reported an 8% failure rate in 23 low-risk patients when oral intubation was performed via the wire-based catheter [20].

### Limitations of the study

Several limitations need to be mentioned. Foremost, several patient data sets were tainted with missing data. For example, 16 (8%, Table 2) of all catheters were removed by the patients themselves without giving us the possibility to check fiberoptically for correct position. Finally, the power of the study was too low, as the null hypothesis could not be rejected. To find a difference between the dislocation rates of 7.2% vs. 2.6%, inclusion of over 600 patients would have been necessary, almost four times more than was anticipated. Furthermore, patients were not randomized, instead the attending anesthesiologist or the requirements of the surgical procedure decided about the placement of either an oral or a nasal AEC.

On the other hand, the combined assessment of waveform capnography and fiberoptic visualization of the correct or incorrect tube position in the PACU was never reported before. This allowed to calculate positive and negative predicted values for the use of capnography to verify correct tracheal position of the exchange catheters.

## Conclusions

As a conclusion, this prospective evaluation of airway exchange catheters in the PACU revealed no statistically significant difference in dislocation rates between nasal and oral placement, but patients with nasal catheters were less prone to retching. In the difficult airway setting, it seems unjustified to exchange oral catheters to a nasal position in order to avoid dislocation. Waveform capnography is insufficient to correctly predict dislocation.
